# Expression of Concern: Lung ultrasound score in establishing the timing of intubation in COVID-19 interstitial pneumonia: A preliminary retrospective observational study

**DOI:** 10.1371/journal.pone.0243267

**Published:** 2020-11-30

**Authors:** The PLOS ONE Editors

Following the publication of this article [1], concerns were raised regarding the similarity between results reported in Table 1 and Table 2 in this article, and results in an article previously published in the *Journal of Intensive Care Medicine* [2]. Specifically,

- The BMI and SOFA scores reported in Table 1 of [1] are identical to the BMI and SOFA scores reported in Table 1 of [2] despite describing different study populations.
- The reported *P* value for the gender division parameters in Tables 1 of both articles [1, 2] is identical.
- The Respiratory rate and the PaCO_2,_ mmHg scores reported in Table 2 of [1] are identical to the T1 (Initial EICU presentation 2 hours) Respiratory rate and the PaCO_2,_ mmHg scores reported in Table 2 of [2] despite describing different study populations.
- The Pulse rate reported in Table 2 of [1] is more similar to the Pulse rate reported in Table 2 of [2] than would be expected from independent studies.

The corresponding author agrees there are similarities between the data reported in these articles [1, 2] and indicated they are checking the underlying data.

PLOS ONE is currently reassessing the article and following up on the above issues in accordance with COPE guidance and journal policies. Meanwhile, the PLOS ONE Editors issue this Expression of Concern.
